# Supplementary material for: Sadness, hopelessness and suicide attempts in bullying: Data from the 2018 Iowa youth survey
Source: PLoS One. 2023 Feb 15;18(2):e0281106. doi: 10.1371/journal.pone.0281106 (PMC9931156; doi:10.1371/journal.pone.0281106)
Supplement: Attachment — Submitted filename: Response to Reviewers.docx [file pone.0281106.s001.docx]

Editor/Reviewer Comments and our responses follow:

Editor: Have the authors made all data underlying the findings in their manuscript fully available?

Response: We are contractually unable to comply with this request. Data for the IYS is collected by the State Departments of Education and Public Health. The data is cleaned and tabulated by the Iowa Consortium for Substance Abuse Research and Evaluation at the University of Iowa, and the data remains the property of the Department of Public Health and considered confidential under State of Iowa, Department of Public Health Disclosure of Confidential Public Health Information, Records, or Data Policy#CO 01-16-002. The publicly available report is available from <https://iowayouthsurvey.idph.iowa.gov/>. See also reference 18.

Reviewer #1: The authors claim to have carried out an ecological study (see abstract), but the reasons that led the authors to this option are not clear enough.

Response: Upon reflection, we agree with the reviewer and have clarified the abstract and body of the paper, calling this a cross-sectional study.

Reviewer #1: Another important aspect is the reference (line 171) that 70,451 validated responses were available for analysis. What would be validated responses and what were the criteria for this validation? How many unvalidated responses were there in the dataset? What would the potential exclusion of unvalidated responses represent to the dataset?

Response: The methods section has been expanded to describe more fully the data collection and cleaning process. The Department of Public Health provided a cleaned data set. We are unable to determine the criteria by which the Iowa Consortium for Substance Abuse Research and Evaluation at the University of Iowa cleaned the data or if exclusion of the unvalidated responses could have influenced the results or our conclusions.

Reviewer #1: Tables 4, 5 and 6 (pages 15-22) need to be better taken care of. Formatting is hampering the reading and interpretation of results, especially as tables take up many pages. It is also necessary to standardize the decimal places of the p-values. I see it as fundamental that the authors present descriptive statistics (n, %) for each variable tested.

Response: The decimal places for all p-values in Tables 4,5, and 6 have all been standardized to 4 significant digits. Adding descriptive statistics to Tables 4,5, and 6 would have resulted in even more cluttering of the tables. Accordingly, Tables 1 and 2 have been expanded to include the total number of responses, the proportion of students answering each question and the proportion indicating “Yes” or “≥ 1 time”.

Reviewer #1: On the other hand, what stands out most in the manuscript was the strategy for discussing the results. The authors chose to debate subsets of data, segmenting the text and making it difficult to read and understand. This impaired the ability to compare the results with other research, making the interpretation superficial. Again, I emphasize that the study presents important results and a very relevant and urgent object. However, it deserves a thorough review of the text.

Response: We take the reviewer’s point seriously and have essentially re-written the entire discussion to reflect interpretation of the data over simply reporting it.

Reviewer #1: Finally, the authors do not make the data available for verification, merely informing that the data is the property of the Iowa Department of Public Health and can be made available upon request. I suggest that the form of data collection used in this manuscript be specifically informed.

Response: As noted above we are contractually prohibited from making the data set available. Interested parties can contact the Iowa Department of Public Health at <https://idph.iowa.gov/PublicHealthData/data-requests> and request access to the data.

Reviewer #2: Abstract: The authors mentioned that the aim was to "...explore the effects of being involved in bullying...". As this was a cross-sectional study, cause-and-effect cannot be inferred. So I recommend to use words such as "associations" instead of "effects".

Response: The reviewer is correct. All references to “effects” have been replaced with “association(s)”.

Reviewer #2: There is a typo on line 61, Pg 3: Cyberybullying

Response: Thank you. This has been corrected.

Reviewer #2: Would the authors consider reporting the values of the ORs and aORs in the abstract? It would provide information on the size of the association.

Response: ORs have been added. aORs are more variable and likely to differ in significance more than ORs. We felt that reporting aORs in the abstract would be more confusing and have left the wording as “… generally lower than unadjusted ORs.”

Reviewer #2: Introduction, Pg 4 Line 76: Please provide the context of Gan et al.'s study, since 55% is a comparatively high prevalence.

Response: We can only speculate as to why Gans et al. had such a high prevalence. We note that they used different instruments to evaluate bullying and that the most frequent forms of bullying in their study were name calling and teasing (40%). These results are similar to our results of social bullying in 35-39% of respondents.

Reviewer #2: Information from the World Health Organization may be useful: <https://www.who.int/news-room/fact-sheets/detail/youth-violence>.

Response: This has been added as Reference 11.

Reviewer #2: Pg 4, ll. 82-83: Please provide the pg number for direct quotations

Response: We are not sure how to respond to this. The quotation is from a journal article, not a book. Page numbers are not generally included for quotations from articles, to our understanding.

Reviewer #2: Pg. 5, ll. 105-109: A key reference on bullying and suicide attempt is:
Koyanagi, Ai, et al. "Bullying victimization and suicide attempt among adolescents aged 12–15 years from 48 countries." Journal of the American Academy of Child & Adolescent Psychiatry 58.9 (2019): 907-918. Please consider citing it.

Response: This has been cited as reference 2.

Reviewer #2: Pg. 6, l. 123 I think ADHD should be spelled attention-deficit/hyperactivity disorder.

Response: The change has been made.

Reviewer #2: Results: Pg. 30, Figure 1: X and Y axes could be reported in more detail

Response: Upon reflection, we did not feel that figure 1 added very much to the discussion, so it has been eliminated. The subsection headed “Variations in Unadjusted ORs” on page 34 provides a clearer explanation of the point we were trying to make.

Results: Pg. 13, Table 1. In the table, you reported "In the last 30 days, how many times have you bullied someone else at school?" This is for bully behavior. However, I do not see the information for experiencing bullying. Is this information not available? If available, I think it would be important to report in this table. Also, the information of bully-victims would be important as well.

Response: Table 2 has been modified to include the proportion of respondents answering each bullying question and the proportion answering ≥ 1 time. There is no single table in the results that totals up a single number of the number of students who experienced bullying.

Reviewer #2: Discussion: I find a large part of the discussion merely reporting the results. A deeper engagement with the results could involve comparisons with other studies, I suggest that the discussion, esp the first 4 paragraphs of the discussion section be revised extensively.

Response: This comment is similar to that of Reviewer 1. We agree and the discussion section has been largely re-written.

Reviewer #2: I think that referring to the "chicken-and-egg problem" twice in the discussion may not be helpful in putting the study in context. Perhaps citing what other researchers have found, and how they explain the findings using past literature/theory could be more effective in providing context to your findings, i.e., linking the current study results with other extant findings in the US and the world?

Response: We agree with the reviewer. This is not essential to the paper and references to it have been deleted.

Reviewer#2: Again, the discussion in Pg. 34-35 was only a repetition of the results, rather than a meaningful discussion of the findings. A major revamp of the discussion section is required.

Response: See previous responses regarding discussion.
